# Survival Trends in Metastatic Renal Cell Carcinoma Across Therapeutic Eras and Histologic Subtypes

**DOI:** 10.7759/cureus.108024

**Published:** 2026-04-30

**Authors:** Sai Tharun Reddy Gopannagari, Ganisetti Divya, Kesava Manikanta Achuta, Chadi Saad

**Affiliations:** 1 Internal Medicine, Garden City Hospital, Garden City, USA; 2 Medicine, Atal Bihari Vajpayee Institute of Medical Sciences (ABVIMS) and Dr. Ram Manohar Lohia (RML) Hospital, New Delhi, IND; 3 Nephrology, Garden City Hospital, Garden City, USA

**Keywords:** cancer metastasis, immune checkpoint inhibitor, kidney neoplasm, renal cell carcinoma (rcc), survival analysis

## Abstract

Introduction

Immune checkpoint inhibitors have transformed the treatment of metastatic renal cell carcinoma (RCC). Whether these advances translated into population-level survival improvements across histologic subtypes remains uncertain.

Materials and methods

We performed a retrospective cohort study using the Surveillance, Epidemiology, and End Results (SEER) database (2006-2022). Patients older than 20 years with distant-stage RCC were categorized into a targeted therapy era (2006-2015) and an immunotherapy era (2016-2022). Overall survival (OS) and cancer-specific survival (CSS) were evaluated using Kaplan-Meier analysis, restricted mean survival time (RMST) analysis (τ=36 months), and multivariable Cox models adjusting for demographic and clinical variables. The interaction between treatment era and histologic subtype was assessed. Sensitivity analyses were restricted to clear cell RCC.

Results

Among 7,356 patients, 5,814 (79.0%) were diagnosed during the targeted therapy era and 1,542 (21.0%) during the immunotherapy era, median OS improved from 13 months (95% CI: 13-14) to 17 months (95% CI: 15-19), and median CSS improved from 14 months (95% CI: 14-15) to 19 months (95% CI: 17-21) (both p<0.001). Diagnosis during the immunotherapy era was associated with improved OS (HR: 0.80, 95% CI: 0.76-0.86) and CSS (HR: 0.77, 95% CI: 0.73-0.83). At 36 months, RMST demonstrated absolute gains of 1.82 months for OS and 1.99 months for CSS (both p<0.001). Survival benefit was attenuated in papillary and chromophobe RCC but remained consistent in clear cell RCC.

Conclusions

Diagnosis during the immunotherapy era was associated with survival improvement in metastatic RCC, predominantly driven by clear cell histology, reinforcing the need for subtype-specific approaches in non-clear cell RCC.

## Introduction

Renal cell carcinoma (RCC) is a heterogeneous group of malignancies originating from the epithelial cells of the renal tubules and accounts for the vast majority of primary kidney cancers in adults [1]. In the United States, approximately 81,800 new cases of kidney and renal pelvis cancers are diagnosed annually, resulting in more than 14,000 deaths and ranking among the most common malignancies in both men and women [2,3]. Although national incidence estimates often group kidney and renal pelvis cancers together, the present study focuses specifically on metastatic RCC.

The most frequent histological subtypes of RCC include clear cell RCC, papillary RCC, and chromophobe RCC, which together account for more than 90% of all RCCs [4]. These subtypes differ in molecular alterations, clinical behavior, prognosis, and therapeutic responsiveness. A substantial proportion of patients present with or progress to metastatic RCC, which historically carries a poor prognosis. Before the introduction of vascular endothelial growth factor (VEGF)-targeted therapies, median overall survival (OS) for patients with metastatic RCC was typically 6-12 months, with outcomes varying according to prognostic factors such as performance status, metastatic burden, metastatic sites, disease-free interval, tumor stage, grade, and histologic subtype, and population-based data continue to demonstrate low five-year cancer-specific survival (CSS) among patients with distant-stage disease [5,6].

Early systemic therapy efforts centered on cytokine-based regimens such as interferon-α and high-dose interleukin-2. Although durable complete responses occurred in a minority of patients, overall response rates were low, and treatment-related toxicity was substantial [7]. The introduction of VEGF-targeted therapies marked a major advancement in the treatment of metastatic RCC. Tyrosine kinase inhibitors such as sunitinib significantly improved progression-free survival compared with cytokine-based therapy and became the standard first-line treatment for advanced RCC throughout the late 2000s and early 2010s [8].

The advent of immune checkpoint inhibitors (ICIs) has transformed first-line therapy for metastatic RCC. Randomized phase III trials demonstrated that ICI-based combinations significantly improved OS compared with VEGF-targeted monotherapy, leading to widespread adoption of immunotherapy regimens [9]. While clinical trials have established efficacy, population-level survival improvements following the widespread adoption of immunotherapy remain less well-defined. Furthermore, the magnitude of benefit across histologic subtypes, particularly papillary and chromophobe RCC, requires further clarification. We therefore evaluated survival trends among patients with distant-stage RCC using a large population-based registry and assessed whether survival improvements in the immunotherapy era vary by histologic subtype.

This work was accepted for poster presentation at MSUCOM Research Day 2026 in Novi, Michigan, USA.

## Materials and methods

Data source

We conducted a retrospective cohort study using data from the Surveillance, Epidemiology, and End Results (SEER) Program, specifically the incidence - SEER Research Data, 17 Registries, November 2024 Submission (2000-2022), released April 2025 [10]. The SEER 17 registries capture approximately 26.5% of the US population and provide information on cancer incidence, tumor characteristics, initial treatment, and survival outcomes. The database was accessed using SEER*Stat software. SEER data are publicly available and de-identified; therefore, institutional review board approval was not required.

Study population

Patients older than 20 years diagnosed between 2006 and 2022 with distant-stage RCC were identified. This cutoff was selected because age at diagnosis was extracted using SEER-categorized age intervals, and “older than 20 years” represented the youngest reliably extractable adult-oriented category available for this analysis. Metastatic disease at diagnosis was defined using SEER Summary Stage 2000 "Distant." Histologic subtypes were identified using ICD-O-3 morphology codes for clear cell, papillary, and chromophobe RCC, as listed in Appendix A.

Treatment era classification

Patients were categorized into two treatment eras based on year of diagnosis: a targeted therapy era (2006-2015), corresponding to the widespread adoption of VEGF-targeted tyrosine kinase inhibitors, and a post-ICI approval era (2016-2022). The 2016 cutoff was selected because nivolumab received FDA approval for advanced RCC in late 2015, making 2016 the first full calendar year after the availability of ICIs in RCC. We acknowledge that first-line ICI combination therapy became established later, particularly after the 2018 approval of nivolumab plus ipilimumab for previously untreated intermediate- or poor-risk advanced RCC.

Variables

Covariates included age at diagnosis (<50, 50-64, 65-74, and ≥75 years), sex (male or female), and race/ethnicity categorized as non-Hispanic White, non-Hispanic Black, non-Hispanic Asian or Pacific Islander, non-Hispanic American Indian/Alaska Native, Hispanic (all races), and non-Hispanic unknown race. Histologic subtype was classified as clear cell, papillary, or chromophobe RCC based on ICD-O-3 morphology codes.

Treatment-related variables included surgical management (no surgery, partial nephrectomy, radical nephrectomy, or other), radiation therapy (none/unknown, external beam, or other radiation), and chemotherapy (yes versus no/unknown), as recorded in SEER.

ECOG performance status, International Metastatic RCC Database Consortium (IMDC) risk score, comorbidity burden, and detailed metastatic burden, including number and sites of metastases, were not available in SEER and therefore could not be included as covariates.

Outcomes

The primary outcome was OS, defined as time from diagnosis to death from any cause. The secondary outcome was CSS, defined as time from diagnosis to death attributed to RCC. Patients alive at the last follow-up were censored at the time of last contact.

Statistical analysis

Survival distributions were estimated using the Kaplan-Meier method and compared using the log-rank test. Multivariable Cox proportional hazards regression models were constructed to estimate adjusted HRs and 95% CIs for OS and CSS, adjusting for age group, sex, race/ethnicity, histologic subtype, surgical management, radiation therapy, and chemotherapy. Proportional hazards assumptions were assessed using Schoenfeld residuals and were satisfied for the overall model and the treatment era.

To evaluate whether the association between treatment era and survival differed by histologic subtype, an additional Cox model was fitted, including a multiplicative interaction term between treatment era and histologic subtype, using the targeted therapy era and clear cell RCC as reference categories. Restricted mean survival time (RMST) analysis was performed with τ=36 months to estimate absolute survival differences between eras. Sensitivity analyses restricted to patients with clear cell RCC were conducted to evaluate the robustness of the findings. All statistical tests were two-sided, and p<0.05 was considered statistically significant. This study followed the Strengthening the Reporting of Observational Studies in Epidemiology (STROBE) reporting guidelines. Analyses were performed using R statistical software (version 4.5.2) (R Foundation for Statistical Computing, Vienna, Austria).

## Results

Study cohort and baseline characteristics

A total of 7,356 patients with metastatic RCC diagnosed between 2006 and 2022 met the inclusion criteria. Of these, 5,814 (79.0%) patients were diagnosed during the targeted therapy era (2006-2015) and 1,542 (21.0%) during the immunotherapy era (2016-2022).

Clear cell RCC was the predominant histologic subtype, comprising 6,633 cases (90.2%). Papillary RCC accounted for 607 (8.3%) cases, and chromophobe RCC for 116 (1.6%) cases. The immunotherapy-era cohort had a modestly higher proportion of older patients, with 16.1% aged ≥75 years, compared with 15.5% in the targeted-therapy era. Racial and ethnic distributions were broadly similar across eras.

Surgical intervention remained common across both eras, although the frequency of radical nephrectomy declined modestly in the immunotherapy era. Radiation and chemotherapy patterns also differed slightly between eras, reflecting evolving treatment practices. Detailed baseline characteristics stratified by era are presented in Table 1.

**Table 1:** Baseline characteristics of patients with metastatic RCC by treatment era (SEER 2006–2022) Values are presented as n (%). RCC, renal cell carcinoma; SEER, Surveillance, Epidemiology, and End Results.

Characteristic	Targeted era (N=5,814)	Immunotherapy era (N=1,542)
Age (years)		
<50	739 (12.7%)	148 (9.6%)
50-64	2,635 (45.3%)	656 (42.5%)
65-74	1,538 (26.5%)	489 (31.7%)
≥75	902 (15.5%)	249 (16.1%)
Sex		
Female	1,780 (30.6%)	471 (30.5%)
Male	4,034 (69.4%)	1,071 (69.5%)
Race		
Hispanic (all races)	1,000 (17.2%)	297 (19.3%)
Non-Hispanic American Indian/Alaska Native	57 (1.0%)	23 (1.5%)
Non-Hispanic Asian or Pacific Islander	389 (6.7%)	92 (6.0%)
Non-Hispanic Black	448 (7.7%)	107 (6.9%)
Non-Hispanic unknown race	10 (0.2%)	5 (0.3%)
Non-Hispanic White	3,910 (67.3%)	1,018 (66.0%)
Histology		
Clear cell	5,265 (90.6%)	1,368 (88.7%)
Papillary	472 (8.1%)	135 (8.8%)
Chromophobe	77 (1.3%)	39 (2.5%)
Surgery		
No surgery	2,503 (43.1%)	717 (46.5%)
Partial nephrectomy	108 (1.9%)	36 (2.3%)
Radical nephrectomy	3,141 (54.0%)	779 (50.5%)
Other	62 (1.1%)	10 (0.6%)
Radiation		
None/unknown	4,011 (69.0%)	1,080 (70.0%)
External beam	1,759 (30.3%)	448 (29.1%)
Other radiation	44 (0.8%)	14 (0.9%)
Chemotherapy		
No/unknown	2,704 (46.5%)	680 (44.1%)
Yes	3,110 (53.5%)	862 (55.9%)

OS

Kaplan-Meier Analysis

**Figure 1:**
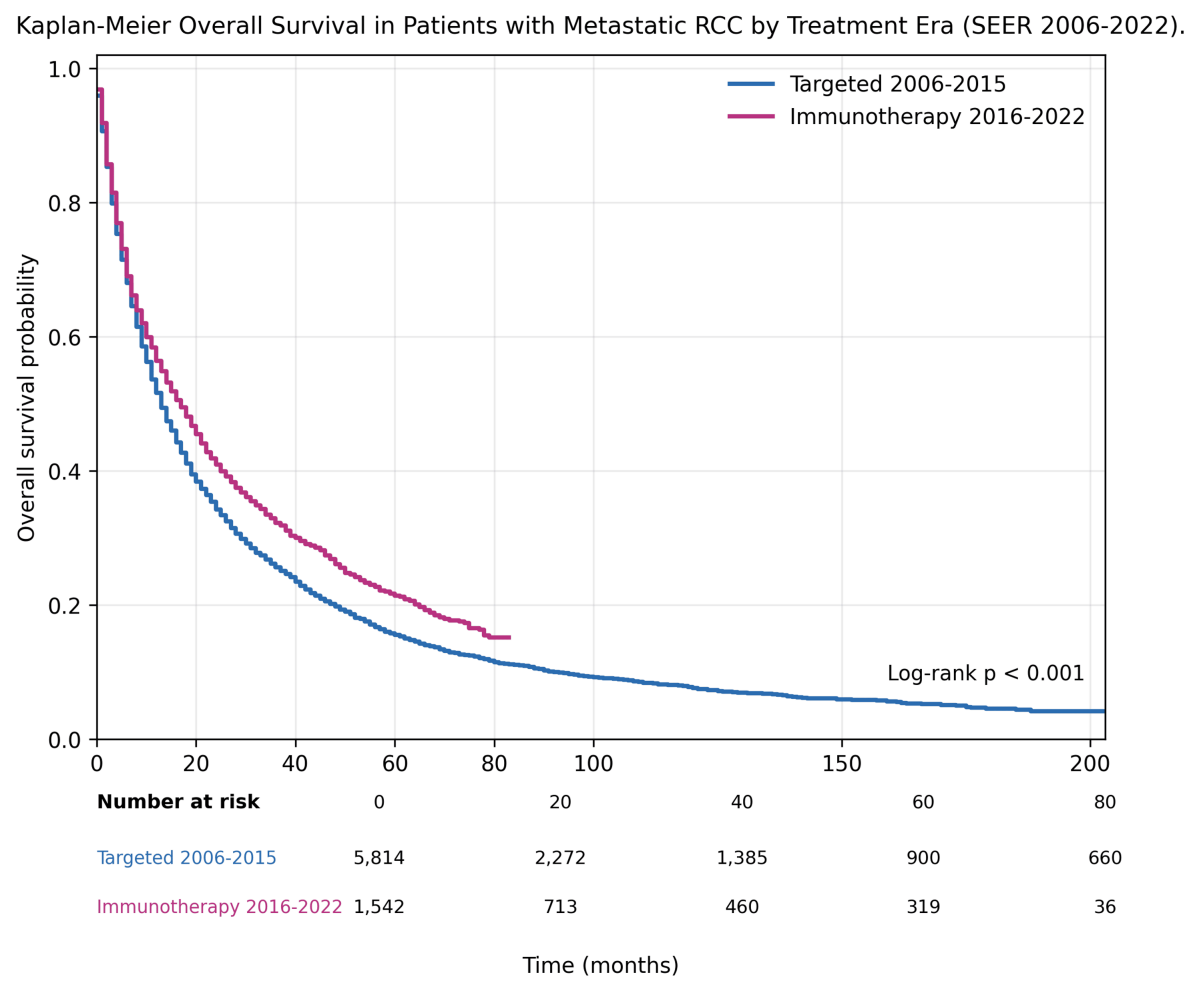
Kaplan-Meier overall survival in patients with metastatic RCC by treatment era (SEER 2006-2022) Survival curves comparing patients diagnosed during the targeted therapy era (2006-2015) and the immunotherapy era (2016-2022). Data are presented as Kaplan-Meier survival probabilities over time. Numbers at risk are displayed at 0, 20, 40, 60, and 80 months. Survival distributions were compared using the log-rank test (χ²(1)=26.853, p<0.001). p<0.05 was considered statistically significant. RCC, renal cell carcinoma; SEER, Surveillance, Epidemiology, and End Results.

Kaplan-Meier analysis demonstrated significantly improved OS in patients diagnosed during the immunotherapy era compared with those diagnosed during the targeted therapy era (log-rank p<0.001). Survival curves separated early after diagnosis and remained persistently divergent throughout follow-up, indicating sustained survival benefit associated with the immunotherapy era (Figure 1).

Median OS was 13 months (95% CI: 13-14) in the targeted therapy era compared with 17 months (95% CI: 15-19) in the immunotherapy era (log-rank p<0.001).

Multivariable Cox Proportional Hazards Model

**Figure 2:**
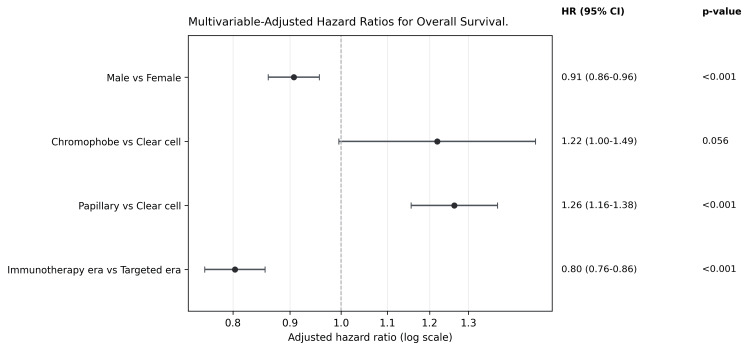
Multivariable adjusted HRs for overall survival Forest plot displaying selected multivariable-adjusted HRs, 95% CIs, and p-values from Cox proportional hazards regression analysis for overall survival among patients with metastatic RCC. HRs less than 1.0 indicate lower mortality risk, whereas HRs greater than 1.0 indicate higher mortality risk. p<0.05 was considered statistically significant. CI, confidence interval; HR, hazard ratio; RCC, renal cell carcinoma.

After adjustment for age group, sex, race/ethnicity, histologic subtype, surgical intervention, radiation therapy, and chemotherapy exposure, diagnosis during the immunotherapy era was associated with improved OS (adjusted HR: 0.80, 95% CI: 0.76-0.86, p<0.001) (Table 2, Figure 2). This corresponds to a 20% relative reduction in mortality compared with the targeted therapy era. The model demonstrated moderate discrimination, with a concordance index of 0.66, indicating that important prognostic factors not captured in SEER may contribute to residual outcome variability.

**Table 2:** Multivariable Cox proportional hazards model for overall survival in patients with metastatic RCC Data are presented as HR with 95% CI. p<0.05 was considered statistically significant. CI, confidence interval; HR, hazard ratio; RCC, renal cell carcinoma; Ref, reference category.

Characteristic	HR	95% CI	p-value
Era			
Targeted era	Ref	—	
Immunotherapy era	0.8	0.76-0.86	<0.001
Age (years)			
<50	Ref	—	
50-64	1.07	0.98-1.16	0.12
65-74	1.13	1.04-1.24	0.004
≥75	1.35	1.22-1.48	<0.001
Sex			
Female	Ref	—	
Male	0.91	0.86-0.96	<0.001
Race			
Non-Hispanic White	Ref	—	
Hispanic (all races)	0.95	0.89-1.02	0.13
Non-Hispanic American Indian/Alaska Native	0.81	0.64-1.03	0.082
Non-Hispanic Asian or Pacific Islander	0.93	0.84-1.03	0.2
Non-Hispanic Black	1.05	0.95-1.15	0.4
Non-Hispanic unknown race	0.68	0.38-1.24	0.2
Histology			
Clear cell	Ref	—	
Papillary	1.26	1.16-1.38	<0.001
Chromophobe	1.22	1.00-1.49	0.056
Surgery			
No surgery	Ref	—	
Other	0.43	0.38-0.49	<0.001
Partial nephrectomy	0.27	0.22-0.33	<0.001
Radical nephrectomy	0.4	0.38-0.43	<0.001
Radiation			
None/unknown	Ref	—	
External beam	1.22	1.16-1.29	<0.001
Other radiation	1.08	0.82-1.42	0.6
Chemotherapy			
No/unknown	Ref	—	
Yes	0.9	0.86-0.95	<0.001

Increasing age was associated with higher mortality. Compared with patients younger than 50 years, those aged 65-74 years had a 13% higher hazard of death (HR: 1.13, 95% CI: 1.04-1.24), and those aged ≥75 years had a 35% higher hazard (HR: 1.35, 95% CI: 1.22-1.48).

Papillary histology was associated with inferior OS compared with clear cell RCC (HR: 1.26, 95% CI: 1.16-1.38, p<0.001). Chromophobe histology demonstrated a trend toward inferior OS relative to clear cell RCC (HR: 1.22, 95% CI: 1.00-1.49, p=0.056).

Surgical management was associated with lower mortality; however, this association likely reflects, at least in part, favorable patient selection. Radical nephrectomy was associated with a 60% reduction in mortality (HR: 0.40, 95% CI: 0.38-0.43), while partial nephrectomy demonstrated an even greater relative reduction (HR: 0.27, 95% CI: 0.22-0.33), compared with no surgery.

External beam radiation therapy was associated with higher mortality risk (HR: 1.22, 95% CI: 1.16-1.29). SEER-recorded chemotherapy exposure was associated with slightly lower mortality (HR: 0.90, 95% CI: 0.86-0.95), although this finding should be interpreted cautiously given the limited granularity of systemic therapy coding in SEER and the potential for misclassification or confounding.

No significant violations of proportional hazards assumptions were observed for the overall model or the treatment era variable. These findings support the validity of the HR estimates over the observed follow-up period.

CSS

Kaplan-Meier Analysis

**Figure 3:**
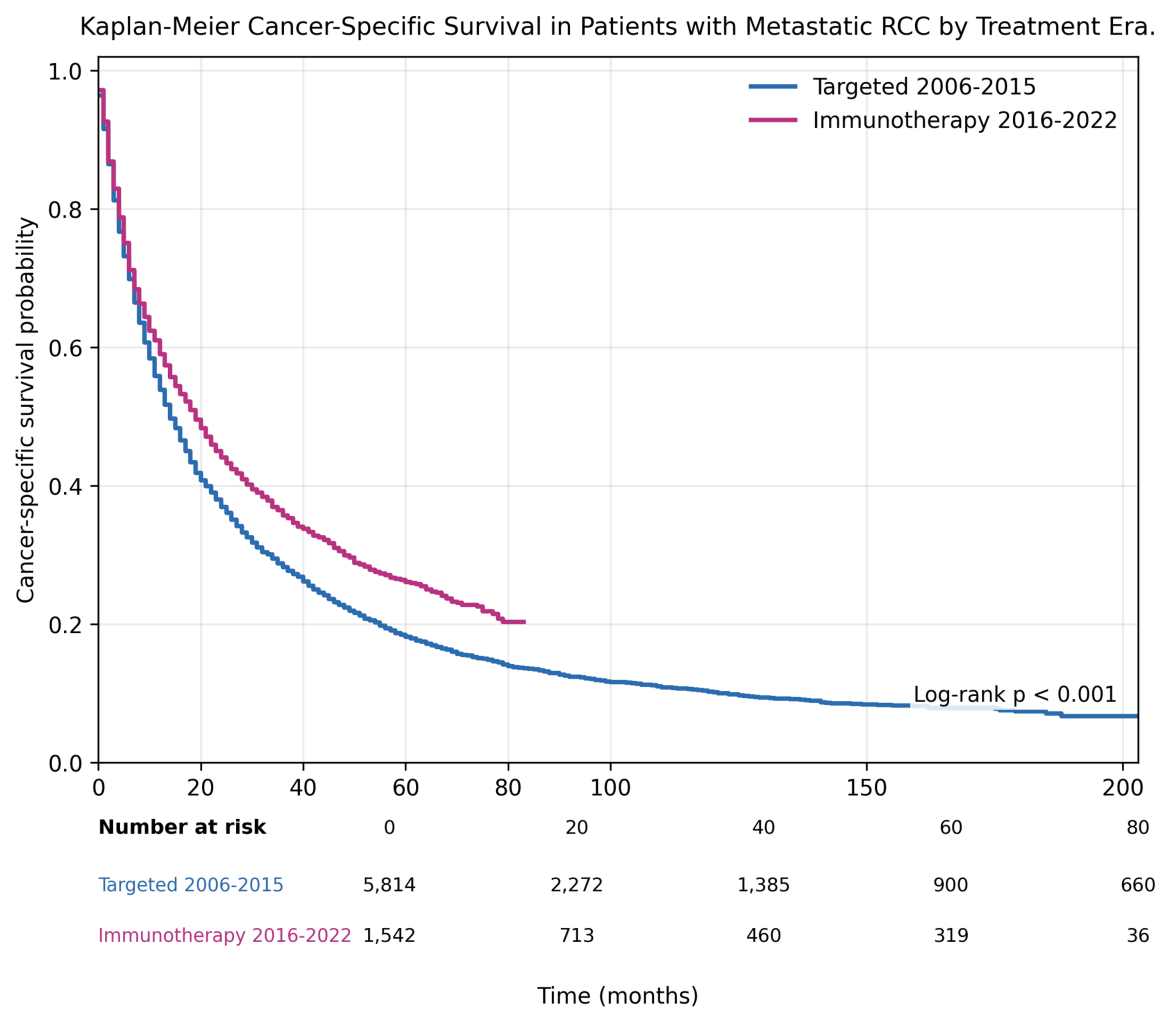
Kaplan-Meier cancer-specific survival in patients with metastatic RCC by treatment era Cancer-specific survival curves comparing the targeted therapy and immunotherapy eras. Data are presented as Kaplan-Meier survival probabilities over time. Numbers at risk are displayed at 0, 20, 40, 60, and 80 months. Survival distributions were compared using the log-rank test (χ²(1)=36.889, p<0.001). p<0.05 was considered statistically significant. RCC, renal cell carcinoma.

CSS analysis similarly demonstrated significant improvement in the immunotherapy era compared with the targeted therapy era (log-rank p<0.001), with persistent separation of survival curves over time (Figure 3).

Median CSS was 14 months (95% CI: 14-15) in the targeted era versus 19 months (95% CI: 17-21) in the immunotherapy era (log-rank p<0.001).

Multivariable Cox Proportional Hazards Model

**Figure 4:**
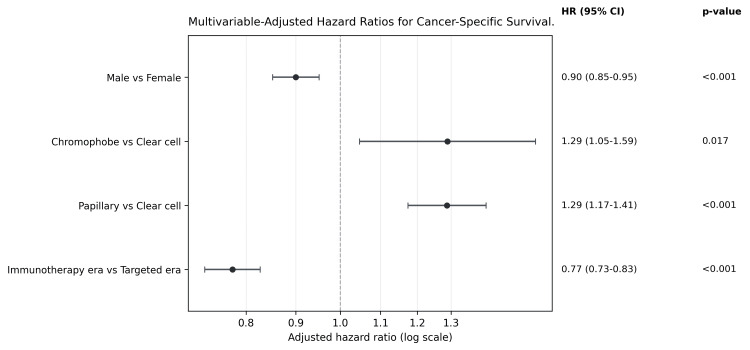
Multivariable adjusted HRs for cancer-specific survival Forest plot displaying selected multivariable-adjusted HRs, 95% CIs, and p-values from Cox proportional hazards regression analysis for cancer-specific survival among patients with metastatic RCC. HRs less than 1.0 indicate lower RCC-specific mortality risk, whereas HRs greater than 1.0 indicate higher RCC-specific mortality risk. p<0.05 was considered statistically significant. CI, confidence interval; HR, hazard ratio; RCC, renal cell carcinoma.

In multivariable analysis, adjusting for the same covariates, diagnosis during the immunotherapy era was associated with improved RCC-specific survival (adjusted HR: 0.77, 95% CI: 0.73-0.83, p<0.001) (Table 3, Figure 4), corresponding to a 23% reduction in cancer-specific mortality.

**Table 3:** Multivariable Cox proportional hazards model for cancer-specific survival in patients with metastatic RCC Data are presented as HR with 95% CI. p<0.05 was considered statistically significant. CI, confidence interval; HR, hazard ratio; RCC, renal cell carcinoma; Ref, reference category.

Characteristic	HR	95% CI	p-value
Era			
Targeted era	Ref	—	
Immunotherapy era	0.77	0.73-0.83	<0.001
Age (years)			
<50	Ref	—	
50-64	1.05	0.97-1.15	0.2
65-74	1.08	0.99-1.19	0.08
≥75	1.26	1.14-1.40	<0.001
Sex			
Female	Ref	—	
Male	0.9	0.85-0.95	<0.001
Race			
Non-Hispanic White	Ref	—	
Hispanic (all races)	0.91	0.85-0.98	0.011
Non-Hispanic American Indian/Alaska Native	0.84	0.66-1.07	0.2
Non-Hispanic Asian or Pacific Islander	0.92	0.83-1.02	0.11
Non-Hispanic Black	0.99	0.90-1.10	0.9
Non-Hispanic unknown race	0.76	0.42-1.38	0.4
Histology			
Clear cell	Ref	—	
Papillary	1.29	1.17-1.41	<0.001
Chromophobe	1.29	1.05-1.59	0.017
Surgery			
No surgery	Ref	—	
Other	0.43	0.37-0.50	<0.001
Partial nephrectomy	0.28	0.23-0.34	<0.001
Radical nephrectomy	0.4	0.38-0.42	<0.001
Radiation			
None/unknown	Ref	—	
External beam	1.25	1.18-1.32	<0.001
Other radiation	1.1	0.83-1.45	0.5
Chemotherapy			
No/unknown	Ref	—	
Yes	0.92	0.88-0.97	0.003

Age-related patterns mirrored those observed in the OS model. Compared with patients younger than 50 years, those aged ≥75 years had a 26% increased hazard of RCC-specific death (HR: 1.26, 95% CI: 1.14-1.40).

Papillary RCC was associated with significantly worse CSS compared with clear cell RCC (HR: 1.29, 95% CI: 1.17-1.41, p<0.001). Chromophobe RCC was also independently associated with inferior CSS relative to clear cell RCC (HR: 1.29, 95% CI: 1.05-1.59, p=0.017).

Surgical intervention remained strongly protective in the CSS model, with radical nephrectomy associated with a 60% reduction in RCC-specific mortality. Associations for radiation and chemotherapy were directionally consistent with those observed in the OS analysis.

RMST Analysis

RMST analysis up to 36 months further supported survival improvement in the immunotherapy era. For OS, the 36-month RMST was 19.11 months (95% CI: 18.40-19.82) in the immunotherapy era compared with 17.29 months (95% CI: 16.94-17.64) in the targeted therapy era, corresponding to an absolute difference of 1.82 months (95% CI: 1.03-2.61, p<0.001). Similarly, the 36-month RMST for CSS was 20.07 months (95% CI: 19.35-20.80) versus 18.09 months (95% CI: 17.73-18.45), yielding an absolute difference of 1.99 months (95% CI: 1.18-2.79, p<0.001). These findings further support absolute survival gains in the contemporary treatment period.

Interaction Between Treatment Era and Histology

Given established biological heterogeneity across RCC subtypes, we formally evaluated the interaction between treatment era and histology in the multivariable OS model.

A statistically significant interaction was identified between era and histologic subtype. Compared with clear cell RCC (reference subtype), the magnitude of survival improvement associated with the immunotherapy era was less pronounced in papillary RCC (interaction HR: 1.31, 95% CI: 1.06-1.61, p=0.012) and markedly reduced in chromophobe RCC (interaction HR: 1.80, 95% CI: 1.17-2.78, p=0.008).

**Figure 5:**
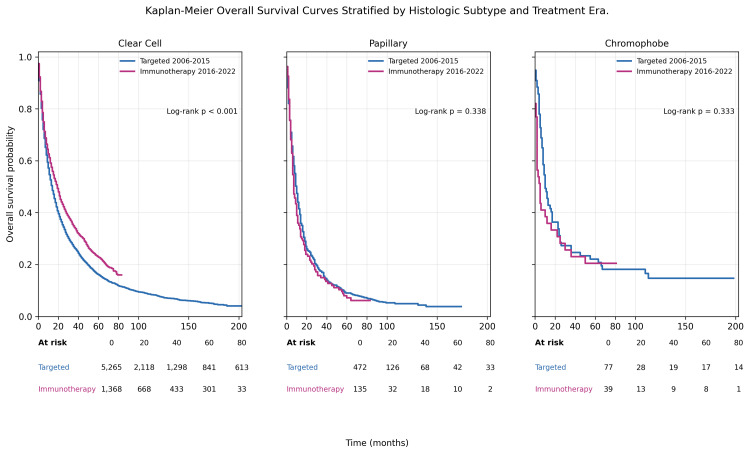
Kaplan-Meier overall survival curves stratified by histologic subtype and treatment era Kaplan-Meier curves demonstrating overall survival among patients with clear cell, papillary, and chromophobe RCC diagnosed during the targeted therapy era (2006-2015) and the immunotherapy era (2016–2022). Data are presented as Kaplan-Meier survival probabilities over time. Numbers at risk are displayed at 0, 20, 40, 60, and 80 months for each histologic subtype and treatment era. Survival distributions were compared using the log-rank test. Among clear cell tumors, overall survival differed significantly by era (χ²(1)=33.934, p<0.001), whereas no significant era-based difference was observed for papillary tumors (χ²(1)=0.919, p=0.338) or chromophobe tumors (χ²(1)=0.938, p=0.333). p<0.05 was considered statistically significant. RCC, renal cell carcinoma.

These findings indicate that the population-level survival gains observed in the immunotherapy era are predominantly driven by patients with clear cell RCC, whereas patients with papillary and chromophobe subtypes derive substantially less benefit. Findings for chromophobe RCC should be interpreted cautiously, given the small subgroup size and the potential for unstable estimates. Kaplan-Meier curves stratified by histologic subtype and treatment era are presented in Figure 5.

Sensitivity Analysis Restricted to Clear Cell RCC

In sensitivity analyses restricted to patients with clear cell RCC (n=6,633; 90.2%), survival improvement in the immunotherapy era remained consistent. Median OS improved from 14 months (95% CI: 13-14) in the targeted therapy era to 19 months (95% CI: 17-21) in the immunotherapy era. In multivariable analysis, diagnosis during the immunotherapy era was associated with a 22% reduction in mortality (HR: 0.78, 95% CI: 0.73-0.83, p<0.001), consistent with the primary analysis.

## Discussion

In this population-based analysis of 7,356 patients with metastatic RCC, we observed statistically significant but modest survival improvements during the post-ICI approval era compared with the targeted therapy era. Median OS improved from 13 to 17 months, and CSS improved from 14 to 19 months, with consistent findings across multivariable Cox regression and RMST analyses. These findings are consistent with the survival benefit demonstrated in pivotal phase III trials such as CheckMate 214 and KEYNOTE-426, which established ICI-based regimens as first-line therapy for advanced RCC [9,11]. Our findings extend these randomized trial results to a broad, unselected US population and confirm that the therapeutic advances of the immunotherapy era have translated into population-level survival gains.

The magnitude of survival improvement observed in our analysis is directionally consistent with randomized phase III data, although somewhat smaller. In CheckMate 214, nivolumab plus ipilimumab demonstrated a significant OS benefit compared with sunitinib. Similarly, KEYNOTE-426 showed improved survival with pembrolizumab plus axitinib relative to sunitinib in the intention-to-treat population [9,11]. Subsequent phase III trials evaluating VEGF-ICI combinations, including CheckMate 9ER and the CLEAR trial, further confirmed superior OS with combination strategies in the first-line setting [12,13]. In contrast, our population-based analysis demonstrated a more modest survival improvement in the immunotherapy era. This difference is expected and likely reflects the heterogeneity inherent in registry-based cohorts, including older patients, greater comorbidity burden, variable treatment uptake, and the absence of risk-stratification data such as the IMDC criteria.

Population-based comparative effectiveness studies have shown improved OS with first-line ICI-based regimens in metastatic RCC, although effect sizes are generally smaller than those reported in randomized trials due to broader patient inclusion and treatment heterogeneity [14,15]. Our study builds on this literature by examining a nationally representative SEER cohort spanning more than 15 years and evaluating both OS and CSS. By formally testing for interaction by histology, we were also able to better characterize which subtypes appear to drive population-level survival gains.

One of the most clinically relevant findings of our study is the significant interaction between treatment era and histologic subtype, demonstrating that survival gains in the immunotherapy era were largely driven by clear cell RCC. While clear cell tumors experienced substantial improvement in OS, papillary and chromophobe subtypes showed attenuated benefit. This observation is biologically plausible, as clear cell RCC is characterized by VHL pathway alterations, angiogenic signaling, and a relatively immunogenic tumor microenvironment, which may enhance responsiveness to VEGF-ICI combinations. In contrast, non-clear cell RCC comprises a heterogeneous group of tumors with distinct molecular drivers and historically lower response rates to immunotherapy [16].

Prospective data in non-clear cell RCC have demonstrated more modest activity of immune checkpoint blockade compared with clear cell populations. For example, recent results from KEYNOTE-B61 reported encouraging but variable response rates in non-clear cell RCC, while the papillary-specific PAPMET trial highlighted the importance of VEGF-targeted strategies in this subgroup [17,18]. Collectively, these findings support the interpretation that immunotherapy-associated survival gains at the population level are predominantly attributable to clear cell disease, and they underscore the persistent unmet need for subtype-directed therapeutic approaches in non-clear cell RCC.

In addition to proportional hazards modeling, we incorporated RMST analysis to quantify absolute survival differences over a clinically relevant time horizon. RMST offers a complementary approach that estimates the average survival time gained within a specified follow-up period and is increasingly recommended as an interpretable alternative to HRs in oncology research [19]. In our cohort, the immunotherapy era was associated with an absolute gain of approximately 1.8 months in OS and 2.0 months in CSS at 36 months, confirming that the observed benefit is not only statistically significant but also clinically meaningful at a population level. The concordance between Cox regression and RMST findings strengthens the consistency of our conclusions. Although statistically significant, the RMST gains observed in this population-based cohort were modest, highlighting the difference between clinical trial efficacy and real-world effectiveness in an unselected registry population.

We also observed that cytoreductive surgery was strongly associated with improved survival, whereas external beam radiation was associated with increased mortality. The apparent protective effect of surgery likely reflects patient selection and favorable disease biology, consistent with ongoing debate following the CARMENA and SURTIME trials, which highlighted the importance of appropriate patient selection in the systemic therapy era [20,21]. In appropriately selected patients with metastatic RCC, surgical tumor removal may retain a meaningful role, as multiple studies have consistently demonstrated an association between cytoreductive nephrectomy and improved outcomes despite inherent selection bias in observational datasets. Conversely, radiation therapy is frequently administered for symptomatic metastatic disease or palliative indications, and its association with worse outcomes in registry data likely reflects confounding by indication rather than a causal relationship. Advanced age was independently associated with increased mortality, consistent with prior epidemiologic observations in metastatic RCC and reflective of comorbidity burden and treatment tolerability considerations in older populations.

This study has several notable strengths. It draws on a large, nationally representative SEER cohort spanning more than 15 years. It captures the transition from targeted therapy to ICI-based regimens in metastatic RCC. By evaluating both OS and CSS and incorporating complementary statistical approaches, including multivariable Cox modeling and RMST analysis, we provide internally consistent evidence of population-level survival improvement. Importantly, the formal assessment of treatment era-by-histology interaction offers novel insight into the differential impact of immunotherapy across RCC subtypes, highlighting that survival gains are predominantly driven by clear cell disease.

Limitations

This study has several limitations inherent to registry-based observational analyses. First, the SEER database does not capture detailed systemic therapy information, including specific immunotherapy agents, treatment combinations, sequencing, line of therapy, or treatment duration. The treatment era was therefore used as a surrogate for evolving therapeutic practices rather than confirmed ICI exposure. Additionally, SEER lacks key prognostic variables such as performance status, comorbidity burden, IMDC risk classification, and comprehensive measures of metastatic burden. Overall disease burden and risk stratification cannot be fully accounted for, raising the possibility of residual confounding. The moderate concordance index of the multivariable model further underscores that key prognostic variables unavailable in SEER may limit risk prediction and should be considered when interpreting the adjusted HRs.

Second, treatment variables, including chemotherapy and radiation, are recorded in binary form and may be misclassified. The association between surgery and improved survival likely reflects both therapeutic benefit and favorable patient selection. Moreover, the era-based comparison is inherently susceptible to temporal bias. Improvements in survival may reflect broader changes in oncologic care over time, including earlier detection of metastatic disease, improved imaging and staging practices, enhanced supportive care, and evolving multidisciplinary management, rather than the effect of immunotherapy alone.

Finally, cause-of-death classification in registry data may be imperfect, potentially influencing CSS estimates. As an observational study, causal inference cannot be established, and findings should be interpreted as associations rather than definitive evidence of treatment effect.

## Conclusions

In this population-based study of metastatic RCC, the immunotherapy era was associated with significant improvements in OS and CSS compared with the targeted therapy era. These gains were consistent across analytic approaches and were largely driven by clear cell histology. The comparatively smaller benefit observed in non-clear cell subtypes highlights the need for histology-specific therapeutic strategies and continued efforts to optimize outcomes in metastatic RCC.

## Appendices

**Table 4:** Appendix A: ICD-O-3 morphology codes used to define RCC histologic subtypes

Histologic subtype	ICD-O-3 morphology code
Clear cell RCC	8310/3
Papillary RCC	8260/3
Chromophobe RCC	8317/3
